# Biomimetic Adhesive Materials Containing Cyanoacryl Group for Medical Application

**DOI:** 10.3390/molecules191016779

**Published:** 2014-10-17

**Authors:** Sueng Hwan Jo, Jeong Sun Sohn

**Affiliations:** 1 Orthopaedic Department, College of Medicine, Chosun University, Gwangju 501-759, Korea; E-Mail: bwjo@chosun.ac.kr; 2 College of General Education, Chosun University, Gwangju 501-759, Korea

**Keywords:** biomimetic adhesive, PEO-PPO-PEO triblock copolymer, coacervate, DOPA, under water adhesive, cyanoacrylate

## Abstract

For underwater adhesives with biocompatible and more flexible bonds using biomimetic adhesive groups, DOPA-like adhesive molecules were modified with cyanoacrylates to obtain different repeating units and chain length copolymers. The goal of this work is to copy the mechanisms of underwater bonding to create synthetic water-borne underwater medical adhesives through blending of the modified DOPA and a triblock copolymer (PEO-PPO-PEO) for practical application to repair wet living tissues and bones, and in turn, to use the synthetic adhesives to test mechanistic hypotheses about the natural adhesive. The highest values in stress and modulus of the biomimetic adhesives prepared in wet state were 165 kPa and 33 MPa, respectively.

## 1. Introduction

Recently, new generations of bioadhesives have been considered for bone fixation, thus anatomically fixating small fragments and avoiding further surgical operations to remove the mechanical devices [1,2,3,4,5,6]. Nevertheless, although many challenges have been undertaken, still no bone adhesives are suitable for practical use in clinical practice [7]. An appreciable model for underwater adhesion is the mussel protein, which is well known for its competence to stick to wet surfaces [2,6,7,8,9,10,11,12].

Mussels release specialized adhesive proteins containing catecholic amino acid, 3,4-dihydroxy-L-phenylalanine (DOPA), which is responsible for both strong interfacial binding and curing of these proteins [2,7]. DOPA is an amino acid that is believed to be responsible for the adhesive characteristics of underwater adhesive proteins (UAPs). The biomimetic approach based on DOPA is to improve adhesive deliverability and performance in the presence of water.

Recently, Messersmith group reported that the catechol form of DOPA bonds to wet titanium oxide surfaces with dissociation energies of 22 kcal/mol, the strongest noncovalent bond yet measured, providing support for DOPA’s main role in interfacial adhesion [13]. Along with exhibiting fine adhesion to polymer surfaces and inorganic metals, DOPA-containing polymeric molecules have been found to strongly interact with a pig gastric glycoprotein in dilute solution, suggesting that DOPA-containing proteins and biomimetic polymers may have useful biocompatible adhesive properties which can be exploited for medical applications [14,15,16,17].

Despite these advantages, underwater bioadhesive (UWB) materials that are currently available rarely meet all the requirements for practical applications due to extremely limited manufacturing, poor adhesion to wet tissues and toxicity concerns. The challenge is to develop effective adhesives for repair of wet living tissues and bones with reasonable production methods.

Bond strengths of barnacle and sandcastle worm (Phragmatopoma californica) glues or byssal thread and plaque assemblies created by mussels (Mytilus edulis) on glass were in the range of 0.2–0.3 MPa [18,19]. These values in underwater bond strengths of bioadhesives are not good enough for practical application. Mimetic adhesives for useful technology should fulfil higher bond strengths at wet state than the ordinary adhesives to find broad practicality.

There have been several approaches to overcome the problems in practical applications of UWB. Of particular interest is the chemical modification of DOPA [20,21,22,23,24,25,26,27,28,29,30]. In an effort to take advantage of the adhesive properties of DOPA, many researches have been focused on the synthesis and characterization of DOPA-containing polymers [21,22,23,24,25]. However, the cross-linking of the DOPA-containing compounds was typically performed by oxidation of DOPA moiety to form DOPA-quinone, which engaged in intra/intermolecular curing reactions to produce a gel network.

The oxidized DOPA is considered to be less adhesive than the unoxidized DOPA [16]. DOPA-containing proteins and polymers reveal better adhesion to metallic wet surfaces when DOPA moiety is not oxidized [23,26]. In this study, an alternative method of coacervate formation that can preserve the unoxidized adhesive form of DOPA was studied. This approach is to conjugate polyethyleneoxide-polypropyleneoxide-polyethyleneoxides block copolymers (PEO-PPO-PEO) with biological moiety that are known to possess desirable adhesive properties in nature, such as DOPA. In literatures reported, low-molecular-weight epoxy resins have been mixed with amphiphilic block copolymers such as poly(ethylene oxide)-block-poly(ethylene-propylene) [22,23]. The initially short epoxy resin is miscible in the higher molecular weight PEO block due to their matching polarity. The triblock architecture could allow an anchoring of the potentially repulsive PPO hydrophobic domains, based on the hydrogen bonding of the PEO blocks to the cathecols of DOPA on both sides. PPO basically induces hydrogen bonds with phenolics and thus it may not be sufficiently repulsive [23] so they could form coacervate hydrophobic/hydrophilic hybrid domain. 

Thus, the aim of this work is to copy the mechanisms of underwater bonding to create synthetic water-borne underwater medical adhesives through the blending of modified DOPA and triblock copolymer (PEO-PPO-PEO), and in turn, to use the synthetic adhesives to test mechanistic hypotheses about the natural adhesive.

## 2. Results and Discussion

### 2.1. Synthesis of Dopamine Methacrylamide (DMA)

**Figure 1:**
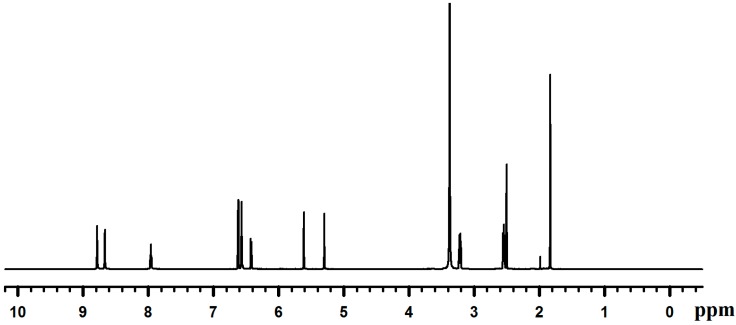
^1^H-NMR spectrum of Dopamine Methacrylamide (DMA).

**Figure 2:**
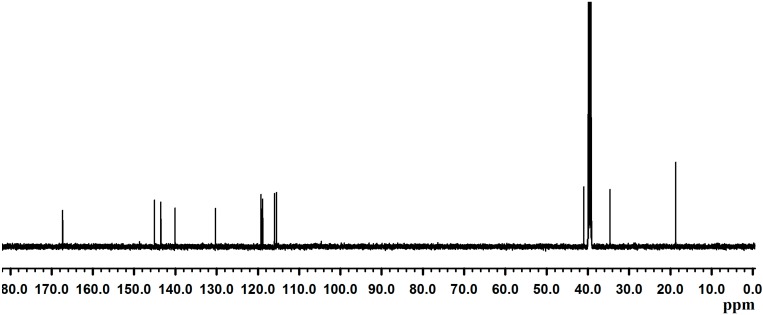
^13^C-NMR spectrum of DMA.

Dopamine-HCl was reacted with methacrylate anhydride to produce DMA with 61% in yield. The prepared DMA was characterized by ^1^H-NMR (Figure 1) and ^13^C-NMR (Figure 2). DMSO-*d*_6_ and TMS were used as a deuterized solvent and an internal standard, respectively, for NMR measurements.

For ^1^H-NMR (300 MHz, DMSO-*d*/TMS): δ 8.7–8.6 [2H, *(OH)*2-Ar-], 7.9[1H, -C(=O)-N*H*-], 6.5–6.6 [2H,C6H*H*2(OH)2-], 6.42[1H, C6H2*H*(OH)2-], 5.61 [1H, -C=O)-C(-CH3)=C*H*H], 5.30 [1H, -C(=O)-C(-CH3)=CH*H*], 3.21[2H,C6H3(OH)2-CH2-C*H*2(NH)-C(=O)-], 2.55 [2H, C6H3(OH)2-C*H*2-CH2(NH)-C(=O)-], 1.84 [3H, -C(=O)-C(-C*H*3)=CH2]. Italic letters indicate the atom taking the peak.

For ^13^C-NMR (300 MHz, DMSO-*d*/TMS): δ 167.3 [s, 1C, -NH-*C*(=O)-C(CH3)=CH2], 145.0 [s, 1C, -NH-C(=O)-*C*(CH3)=CH2], 143.5–115.5 [6C, *C*6H3(O-C(=O)-CH3)2], 130.3 [s, 1C, -NH-C(=O)-C(CH3)=*C*H2], 41.0 [s, 1C, C6H3(OH)2-CH2-*C*H2(NH)-C(=O)-], 34.6 [s, 1C, C6H3(OH)2-*C*H2-CH2(NH)-C(=O)-], 18.7 [s, 1C, -C(=O)-C(-*C*H3)=CH2].

**Figure 3:**
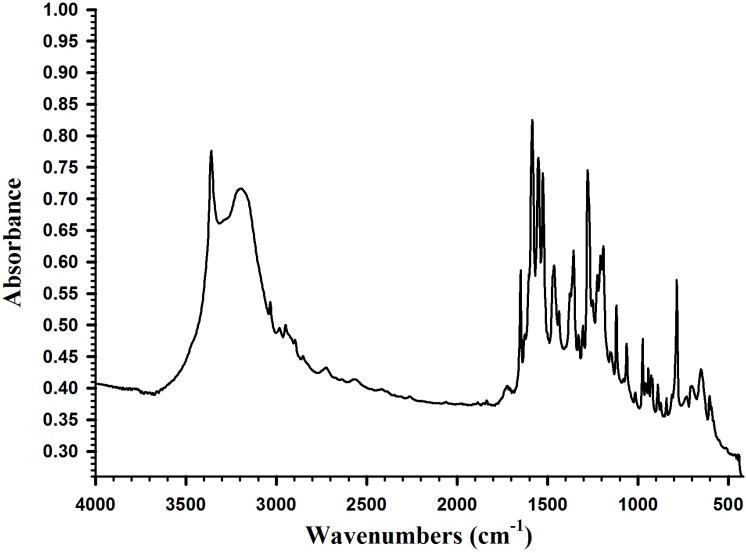
IR spectrum of DMA.

Figure 3 shows the IR absorption spectrum of DMA. The functional groups of DMA were identified at 1650 cm^−1^ (υ_C=C_), 1670 cm^−1^ (υ_C6H5_), 2750 cm^−1^ (υ_OH_), 3250 cm^−1^ (υ-_NH_), and 1560 cm^−1^ (υ_C=O_).

### 2.2. Synthesis of Poly(dopamine methacrylamide-co-Methoxyethyl acrylate), PDM, and Poly(dopamine methacrylamide-co-Methoxyethyl acrylate-co-2-ethyl cyano acrylate), PDMC

**Scheme 1:**
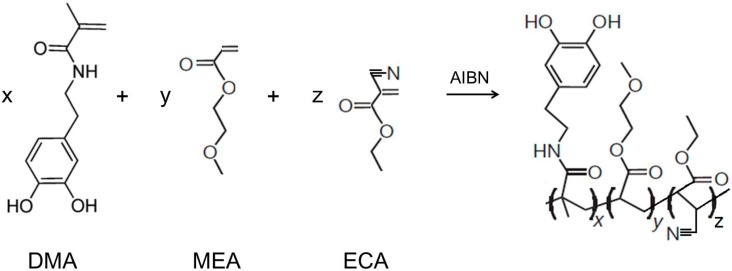
Synthetic scheme of poly(DMA-co-MEA) and poly(DMA-co-MEA-co-ECA) from DMA + MEA + ECA.

Notes: DMA: dopamine methacrylamide; MEA: 2-methoxyethyl acrylate; ECA: ethyl 2-cyanoacrylate; PDM: poly(DMA-MEA), x = y = 0.5, z = 0.0; PDMC: poly(DMA-MEA-ECA), x = y = 0.5, z = 1.0.

PDM was synthesized by free radical copolymerization of the DMA and MEA monomers with AIBN as a radical initiator as shown in Scheme 1. The monomer feed ratio of DMA and MEA was adjusted to 74 mmol (DMA) and 60 mmol to produce 1 to 1 monomer unit contents in the final polymer composition according to several prior trials.

**Figure 4:**
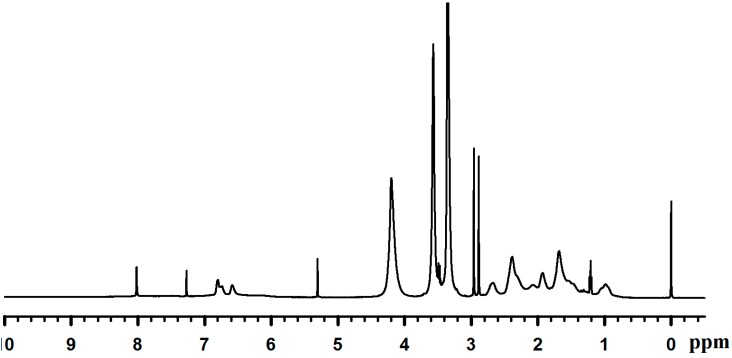
^1^H-NMR spectrum of Poly(dopamine methacrylamide-co-Methoxyethyl acrylate), PDM.

^1^H-nuclear magnetic resonance spectrum (300 MHz) was obtained with CDCl_3_ as a solvent and TMS as an internal standard (Figure 4).

1H: 6.81–6.70 (d, br, 2H, C6H*H*2(OH)2-), 6.58 [s, br, 1H, C6H2*H*(OH)2-], 4.20 [s, br, 2H, CH3-O-CH2-C*H*2-O-C(=O)-], 3.57 [s, br, 2H, CH3-O-C*H*2-CH2-O-C(=O)-], 3.36 [s, br, 3H, C*H*3-OCH2-CH2-O-C(=O)-], 2.69 [s, br, 2H, C6H3(OH)2-CH2-C*H*2 (NH)-C(=O)-], 2.39 [s, br, 1H, -O-C(=O)-C*H*(CH2-)-CH2-], 2.14 [s, br, 2H, C6H3(OH)2-C*H*2-CH2(NH)-C(=O)-], 1.93 [s, 3H, -NHC(=O)-C(CH3)(CH2-)-CH2-], 1.68 [m, br, -O-C(=O)-CH(CH2-) -C*H*2-], 0.98 [m, br, -NH-C(=O)-C(CH3)(CH2-)-C*H*2-]. This NMR analysis indicated a 1:0.9 molar ratio of DMA to MEA in the copolymer composition.

**Figure 5:**
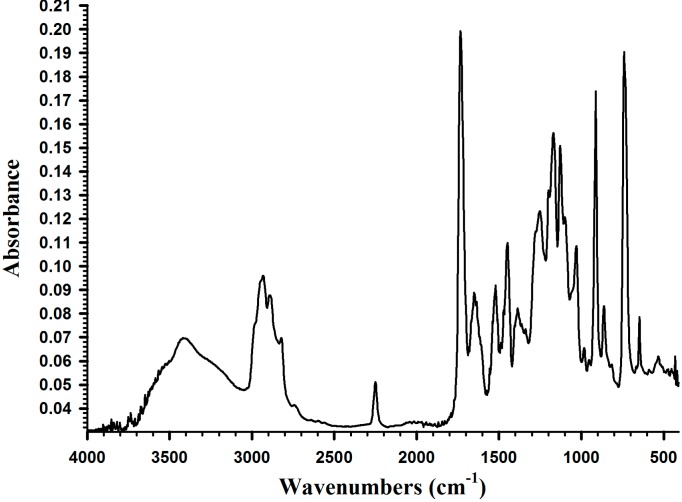
IR spectrum of PDM.

IR spectrum of PDM gives a distinct hydroxyl group of cathecol group of DOPA unit at 2931 cm^−1^ for cathecol OH and carbonyl C=O at 1732 cm^−1^ (Figure 5).

**Figure 6:**
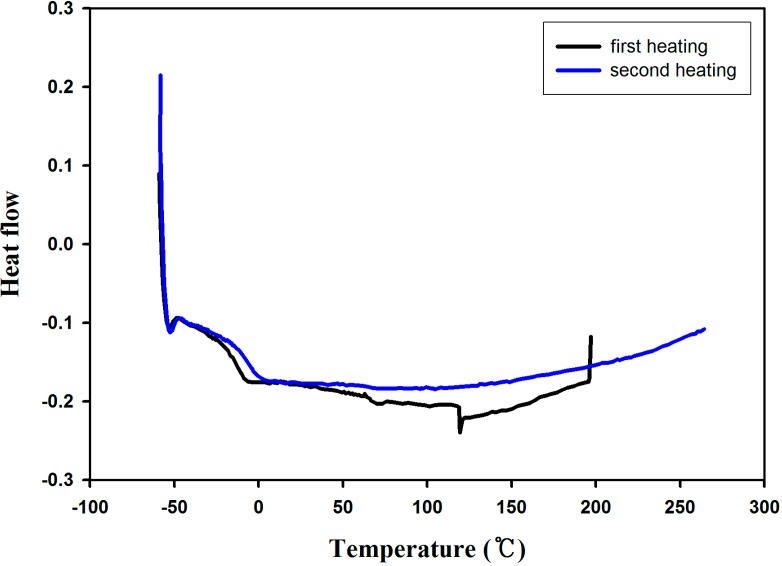
Differential scanning calorimetry diagram of PDM.

The yield of PDM was 65% and the average Mn was 12,000 g/mol. The glass transition was observed at −15 °C on the 1st and 2nd heating runs of differential scanning calorimetry as shown in Figure 6.

**Figure 7:**
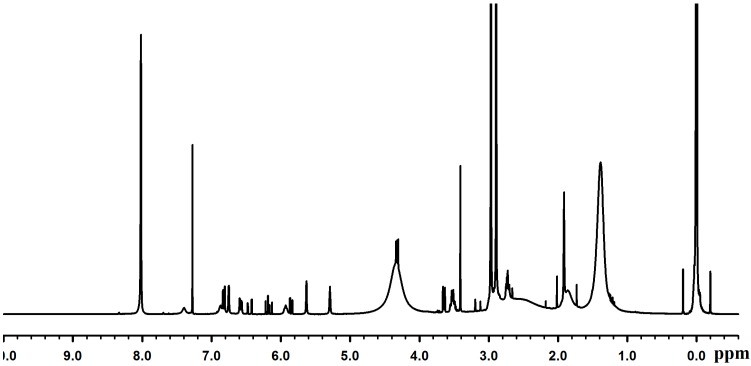
^1^H-NMR spectrum of Poly(dopamine methacrylamide-co-Methoxyethyl acrylate-co-2-ethyl cyano acrylate), PDMC.

**Figure 8:**
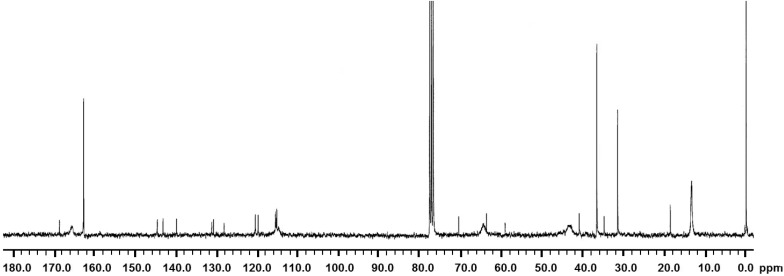
^13^C-NMR spectrum of PDMC.

**Figure 9:**
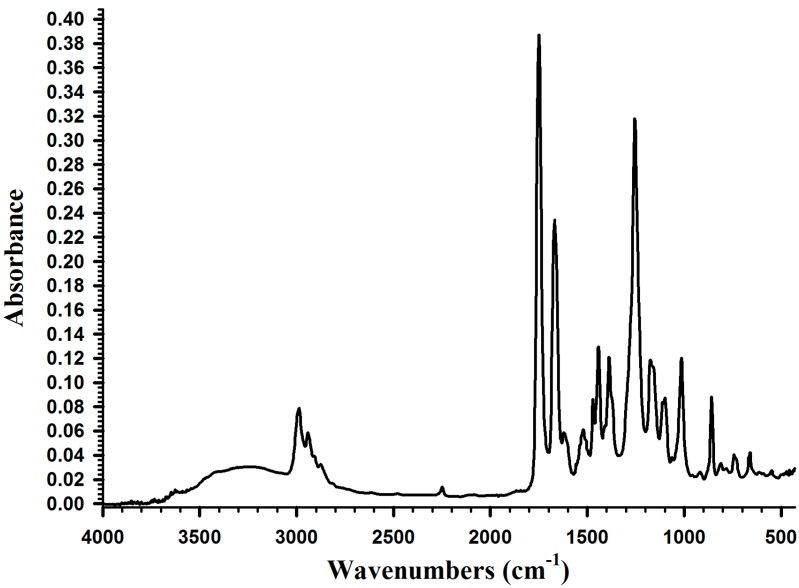
IR spectrum of PDMC.

PDMC was synthesized by a thermally initiated free radical polymerization of DMA, MEA and ECA as shown in Scheme 1. The prepared polymer was characterized by ^1^H-NMR to calculate composition ratios of each repeating unit in the final polymer (DMA-co-MEA-co-ECA). ^1^H-NMR and chemical structure assignments are shown in Figure 7. After testing, samples which were prepared with various ratios of three repeating units, a tricopolymer which has 25% of DMA, 25% of MEA and 50% of ECA, demonstrated the most suitable properties for use as an adhesive. Therefore, this report focuses on an adhesive of this composition. According to ^1^H-NMR characterization (Figure 7), the proton of CH-CN showed at 3.4 ppm that the final composition reflects the initial feeds of monomers. ^13^C-NMR gives a distinct carbon of CN group in the PDMC at 162.6 ppm (Figure 8). IR spectrum of PDMC showed CN stretching vibration at 2040–2060 cm^−1^ (Figure 9). The yield of the PDMC was 71% and the average Mn was 7500 g/mol.

**Figure 10:**
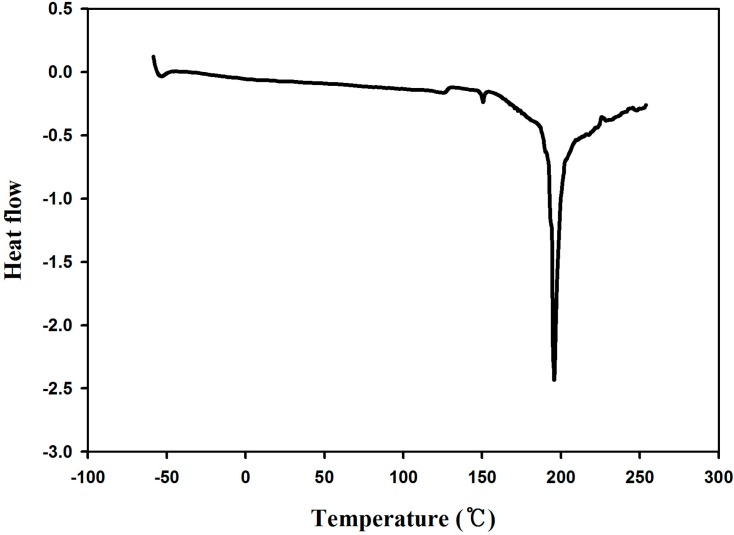
Differential scanning calorimetry diagram of PDMC.

The melting transition was observed at 198 °C with quite crystalline morphology on the heating run of differential scanning calorimetry as shown in Figure 10.

### 2.3. Adhesion Properties

**Figure 11:**
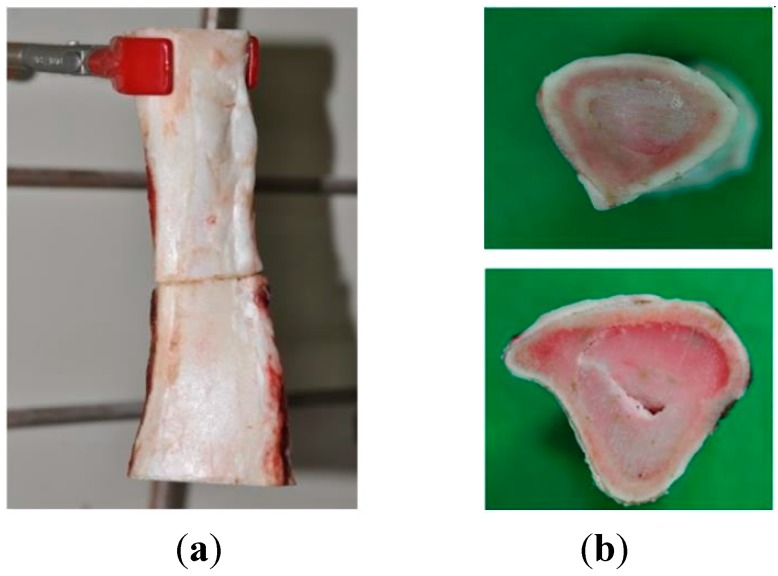
Adhesion of wet bone by coacervates: vertical arrangement after adhesion (**a**) and specimen surface (**b**).

The mechanical properties for adhesion such as shear modulus and stress at break were measured using universal testing machine with the bone specimens adhered while wet (Figure 11). Super Glue was used as a control because there are no bone adhesives in clinical use for comparison. Adhesion tests were performed on stainless steel plates and bone specimen with the coacervates in five different compositions as described in Table 1, that is, CC-1; PDM with EPE (PEO-PPO-PEO tri-block polymer) in 1:1 ratio, CC-2; PDMC with EPE in 1:1 ratio, CC-3; PDM and PDMC with EPE in 1:1:1 ratio, CC-4; PDM and PDMC with EPE in 1:2:1 ratio and CC-5; same as CC-4 but no Ca^2+^/Mg^2+^ content. All surfaces were handled according to the same procedure.

Adhesion properties such as stress at break, modulus and contact angle of the sample specimens were identified as shown in Table 2.

**Table 1:** Compositions of the coacervates in weight portion.

CC	PDM	PDMC	EPE	Ca^2+^/Mg^2+^
CC-1	1	0	1	0.1
CC-2	0	1	1	0.1
CC-3	1	1	1	0.1
CC-4	1	2	1	0.1
CC-5	1	2	1	0.0

**Table 2:** Shear adhesion properties of complex coacervates on stainless steel plaques.

CC	Stress at Break (kPa)	Modulus (MPa)	Contact Angle (deg)
CC-1	75 ± 6.8	18 ± 2.5	76 ± 2.3
CC-2	96 ± 10.5	40 ± 5.2	87 ± 4.3
CC-3	138 ± 15.6	27 ± 3.2	95 ± 3.7
CC-4	165 ± 13.5	33 ± 3.5	110 ± 3.5
CC-5	125 ± 10.5	28 ± 3.0	110 ± 5.8
Super Glue	275 ± 3.5	42 ± 4.8	116 ± 6.9

For adhesion in general, force is related to overlap area, with greater area yielding higher binding forces [31]. Because of the problems in determining the exact contact area, the values in modulus (shear) were taken from the tests in stainless-steel adhesion and, on the other hand, the values in stress at break were from the bone specimen tests. The columns of stress at break and modulus were each averaged from six individual specimens. The average adhesion varies dramatically as expected depending upon the amount of PDMC content. In case of CC-2, for example, its modulus showed 40 MPa which nearly approaches that of Super Glue (42 MPa) but 96 kPa, the value of stress at break, was quite low compared with Super Glue (273 kPa). The water contact angle was measured for each surface, prior to placement in the tanks, and these data are also shown in Table 2. The data provide insight on the relative surface energy of each substrate. CC-1 having PDM and EPE with Ca^2+^ and Mg^2+^ cations showed 76° in contact angle, on the other hand, CC-4 containing PDM, EPE and higher PDMC showed 116° which is more hydrophobic due to higher ECA content. The highest value in stress at break was 165 kPa shown in CC-4 which is about 65% of the strength of wet bones bonded with commercial cyanoacrylate adhesive (Super Glue).

In these complex coacervates, anion-cation interactions could contribute to enhanced adhesive bonding between the plaques and surface. For example, the CC-5 containing the same polymer content with CC-4 but no Ca^2+^ and Mg^2+^ cations showed 125 kPa in stress at break and 28 MPa in modulus. These values are quite low compared with CC-4 which means that anion–cation interactions especially have a stronger effect on the adhesion strength and modulus. Both the modulus and stress at break of the fully hydrated specimens increased with increasing divalent cation concentration. The coacervate with no Mg^2+^/Ca^2+^ resulted in the weaker bond. Other chemical contributions to strong adhesion on stainless-steel may include hydrogen bonding between the coacervate and the metal oxide surface as well as chelation of surface metal ions by the 3,4-dihydroxyphenylalanine (DOPA) of the polymers PDM and PDMC. The bonds were quite stable, neither swelling nor shrinking apparently after complete immersion in PBS (10 mM PO_4_, 120 mM NaCl, pH 7.2) for several weeks. Dimensional stability during cure and long exposure to physiological ionic strength and pH is an important requirement for a useful bone adhesive. 

**Figure 12:**
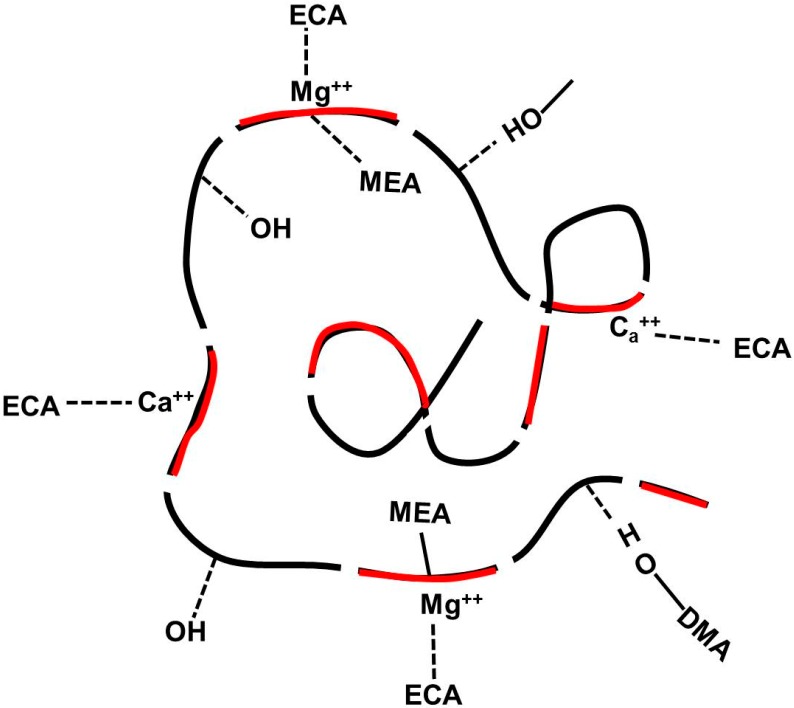
Model of coacervate structure and adhesive mechanisms.

Figure 12 gives a model of coacervate structure and adhesive mechanisms. A: PDM/ PDMC and PEO(black)-PPO(red)-PEO block with Ca^2+^ and Mg^2+^ form nm-scale complex domain in water. The coacervate can adhere to the bone surface through electrostatic interactions, 3,4-dihydroxyphenol side chains, and quinone-mediated covalent coupling to matrix proteins. In this context, poly(ethyleneoxide)-poly(propyleneoxide)-poly(ethyleneoxide)s, PEO-PPO-PEO, triblock copolymers, are of significant interest because they exist in different states of aggregation in aqueous solution depending on relative block sizes and hydrophilicity. Their self-association behaviour in water has attracted great attention in the literature [20,27,28].

The coacervate liquid has low initial viscosity (η_inh_ 0.85) and specific gravity greater than 1 (1.15), all of which contribute to its activity to wet the bone surface. The coacervate adheres to moist bone shred and other inorganic materials through multiple mechanisms. The surface of the bone’s hydroxyapatite portion [Ca_5_(PO_4_)_3_(OH)] displays an arrangement of both positive and negative charges. The polyphosphate anion can interact directly with the surface cation or it can be bonded physically to the negative surface charges via the positive polyamine and/or the divalent cations. In the same way, charge–charge interaction of the polyamine with the surface bridged to the negative charges through the polyamine and/or divalent cations thereby contribute to adhesion [25]. Molecules containing catechol portions have been revealed to have strong absorptivity and to easily wet the bone surface.

## 3. Experimental Section

### 3.1. Materials

Triblock poly(ethylene oxide)-poly(propylene oxide)-poly(ethylene oxide), PEO-PPO-PEO (Pluronic F-127 as a trade name), average molecular weight of 12,600 g/mol (oxyethylene content, 71.5%–74.9%, content of PEO 80%) and an inhibitor-removing column, Sephadex LH-20 were purchased from Sigma-Aldrich Co. and used without further treatment. Methoxyethyl acrylate (MEA), 2-ethyl cyano acrylate (ECA, 99%, Tm 84–86 °C and azobisisobutyronitrile (AIBN) 97% were purchased from Aldrich Chemical Co. and used as-received. Super Glue, Loctite 401 (Henkel, Dusseldorf, Germany), was used as a control. All other chemicals and organic solvents of the highest purity available were obtained from commercial sources.

### 3.2. Syntheses

#### 3.2.1. Synthesis of Dopamine Methacrylamide (DMA), DOPA Analog Monomer

For synthesis of dopamine methacrylamide (DMA), DOPA analog monomer, Messersmith’s method was employed with slight modification [32]. Sodium borate (20 g) and sodium bicarbonate (8 g) were dissolved in deionized water and bubbled with Ar. 10 g of dopamine-HCl (52.8 mmol) was then added, followed by the dropwise addition of 9.4 mL of methacrylate anhydride (58.1 mmol) in 100 mL of THF to keep the pH above 8 with addition of 1 M NaOH. The reaction mixture was stirred overnight at room temperature with Ar bubbling. The aqueous mixture was washed several times with 300 mL of ethyl acetate and then the pH of the aqueous solution was reduced to less than 2 and extracted with 100 mL of ethylacetate three times. The final three ethyl acetate layers were combined and dried over MgSO_4_ to reduce the volume to around 50 mL. Eight hundred mL of hexane was added with vigorous stirring and the suspension was kept in a refrigerator overnight. The product was recrystallized from cold *n*-hexane and dried to yield 8.8 g of grey solid.

#### 3.2.2. Synthesis of Poly(dopamine methacrylamide-co-Methoxyethyl acrylate), [Poly(DMA-co-MEA)]

Polymer, p(DMA-co-MEA) was synthesized by free radical copolymerization of the DMA and MEA monomers by slight modification of published procedure [32] as shown in Scheme 1 and its molecular weight was analysed by size exclusion chromatography (Waters Technology). MEA was passed through a column packed with Al_2_O_3_ to remove inhibitor. 7.5 g of purified MEA (60 mmol), 17 g of DMA (74 mmol) and 120 mg of AIBN (0.71 mmol) were added to 70 mL of DMF in an airtight flask. The solution mixture was degassed through pump-freeze-thaw cycles three times. While sealed under vacuum, the solution was heated to 65 °C and stirred overnight. The reaction mixture was diluted with 250 mL of methanol and added to 500 mL of methyl ethyl ketone to precipitate the polymer. After precipitating in dichloromethane three times and drying in a vacuum desiccator, 11.2 g of brownish, sticky solid was obtained. Gel permeation chromatography in concert with multi-angle laser light scattering (Wyatt Technology), with mobile phase of 20 mM LiBr in DMF and Shodex-OH Pak columns: weight-average molecular mass 420 kDa, polydispersity 25.2. For control experiments, a catechol-free p(MEA) homopolymer (molecular mass (average) 5100 kDa, Scientific Polymer Products) was used.

#### 3.2.3. Synthesis of Poly(dopamine methacrylamide-co-Methoxyethyl acrylate-co-2-ethyl cyano acrylate), [Poly(DMA-co-MEA-co-ECA), PDMC]

ECA, DMA and MEA were copolymerized in the monomer feed ratio 2:1:1 by free radical polymerization on the same way for poly DMA-co-MEA preparation as shown in Scheme 1. Cyanoacrylate was passed through an inhibitor-removing column to remove the polymerization inhibitor (monomethyl ether hydroquinone).

In a typical synthesis procedure, a mixture of 6.5 g of purified MEA (50.0 mmol), 11.1 g of DMA (50.0 mmol) and 12.5 g of ECA (100.0 mmol) were added to 100 mL of DMF in an airtight flask and 300 mg of AIBN was placed into a borosilicate glass vial covered with a sleeve rubber stopper and equipped with a gas inlet/outlet. The mixture was deoxygenated by nitrogen flow overnight at 10 °C and immersed in oil bath. The bath was heated from 10–60 °C and then kept at 60 °C for 48 h. Then, the vial was allowed to equilibrate at room temperature and the polymer was precipitated in chloroform. The polymer sample produced was dried under vacuum to constant weight.

### 3.3. Polymer Characterization Procedure

The polymers synthesized were characterized by FTIR, ^1^H-NMR and ^13^C-NMR spectroscopy analyses. NMR spectra of the polymers were obtained with a Varian NMR 300 model at a proton resonance frequency of 300 MHz on using CDCl_3_ as a solvent and tetramethylsilane (TMS) as an internal reference. Nicholet FT-IR spectrometer model 6700 was used to evaluate the extent of functional groups. For this purpose, 1 mg of polymer sample was mixed with 100 mg of KBr containing 1 wt percentage of KSCN. The main polymeric fraction, washed with chloroform, was dried under vacuum to constant weight and analyzed by gel-permeation chromatography (GPC) to determine average molecular weight of the prepared polymer. GPC analyses were run on a GPC Waters model 600 E in tetrahydrofuran (THF) on two Waters Ultrastyragel linear columns and in water on two Waters Ultrahydrogel linear columns by using a RI detector.

A drop shape analyser (DSA100S, Kruss, Hamburg, Germany) was used for measuring contact angles of the samples. In this static sessile drop method, a drop was deposited on a surface and the contact angle was measured by looking at the drop through a goniometer.

### 3.4. Preparation of Blends and Complex Coacervates

Blends of poly (DMA-co-MEA)/PEO-PPO-PEO, PDM/EPE or poly (DMA-co-MEA-co-ECA)/PEO-PPO-PEO, PDMC/EPE were prepared in 1:1 ratio each by dissolving the components in ethanol (50 vol %) at their required compositions. The mixtures were stirred for 1 day at room temperature and finally for 10 min at 60 °C, after which they were transparent. Calcium sulfate and Magnesium sulfate (0.5 wt % each) in DI water were added into the above each solution to get a complex coacervate. The compositions of complex coacervates are as Table 1.

### 3.5. Bone Adhesion Property Test

**Figure 13:**
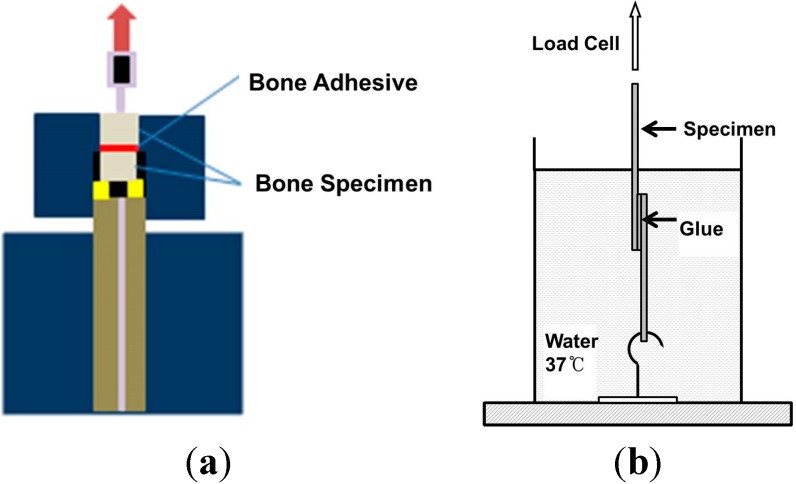
Test of stress at break (**a**) and for shear modulus (**b**).

Stainless steel plates measuring 30 mm × 30 mm were used for shear test of the adhesives (Figure 13a). The plates were cleaned, polished, and glued together by applying a layer of *ca.* 15 mg of the adhesive to the polished face of the adherend. The adherends were tightly pressed for 30 s. The adherends were then allowed to hold for 24 h at ambient temperature. Bone test specimens were cut with a band saw from freshly sacrified bovine femur cortical bone, obtained from a local grocery store (Figure 13b). The shear modulus and strength at failure were measured with the bone specimen bonded while wet. The bone specimen was thoroughly wet by water before applying the adhesive. Bone samples were soaked in PBS buffer (20 mM Phosphate, 150 mM NaCl, pH 7.4) before bonding. The adhesives were prepared at pH 7.2 as using the sample specimens (CC-1, 2, 3, 4 and 5). Ascorbic acid was added at ratio of 1:5 to the polymers to prevent premature DOPA oxidation in the specimens. NaIO_4_ at a 1:2 molar ratio to DOPA side chains was evenly applied onto one face each of two wet bone specimens. A volume sufficient (0.2 g/cm^2^) to completely fill the space between bone interfaces of the test specimen was applied with a pipette. The two pieces of bone specimens were then pressed together, clamped, and immediately wrapped with PBS soaked gauze. All the bonded specimens were incubated in a sealed container containing soaked sponges to maintain sufficient humidity (100%) at 37 °C for at least 24 h. The stainless steel plates were fully submerged in a temperature-controlled water bath at 37 °C during the test (Figure 13a). Super Glue was used as a control. Control specimens were bonded with sufficient amount (0.2 g/cm^2^) of Super Glue in exactly the same manner. A commercial nonmedical grade cyanoacrylate was used because there are no hard tissue medical adhesives available for comparison. The shear mechanical tests were performed on a UTM (Autograph, Shimadzu Co., Tokyo, Japan) with a water jacket and a 1 kg load cell (Figure 13a). One piece of bone of a bonded pair was clamped laterally 1 mm from the bond interface. The other piece was pressed against a dull blade positioned 1 mm lateral to bond interface with a crosshead speed of 0.02 mm/s until failure. Mechanical bond testings were performed in the water jacket at 37 °C as mentioned previously. At least six specimens were tested for each condition. Errors are reported as the standard error of the mean.

## 4. Conclusions

In this study, it concluded that a UAP adhesive created by mimicking the mussel adhesive with a composition of synthetic DOPA-cyanoacrylate containing copolymers and divalent cations with PEO-PPO-PEO triblock copolymer was successfully prepared and characterized.

The sample, CC-4, in wet state gives 165 kPa in stress at break and 33 MPa in modulus which values are good enough for repair of wet living tissues and bones.

Complex coacervates could be an ideal state of matter in material for medical adhesives. Induced by a natural adhesive produced by a marine mussel, we have taken an alternative way to exploit adhesives for orthopedic surgery and the further development of medical applications through biomimetic chemical syntheses and their blending for coacervate complexes.
